# Evaluation of reference genes for normalizing RT-qPCR in leaves and suspension cells of *Cephalotaxus hainanensis* under various stimuli

**DOI:** 10.1186/s13007-019-0415-y

**Published:** 2019-03-26

**Authors:** Huapeng Sun, Xuefei Jiang, Mengli Sun, Hanqing Cong, Fei Qiao

**Affiliations:** 1 Key Laboratory of Crop Gene Resources and Germplasm Enhancement in Southern China, Ministry of Agriculture/Tropical Crops Genetic Resources Institute, Chinese Academy of Tropical Agricultural Sciences, Danzhou, 571737 Hainan People’s Republic of China; 2 Hainan Key Laboratory of Sustainable Utilization of Tropical Bioresources/Institute of Tropical Agriculture and Forestry, Hainan University, Haikou, 570228 Hainan People’s Republic of China

**Keywords:** RT-qPCR, Reference gene, *C. hainanensis*, Expression stability, Normalization

## Abstract

**Background:**

Reverse transcription quantitative real-time PCR (RT-qPCR) is a widely used approach for investigating gene expression levels in plants because of its high reproducibility, sensitivity, accuracy and rapidness. Evaluation of reference genes for normalizing RT-qPCR data is a necessary step, especially in new plant varieties. *Cephalotaxus hainanensis* is a precious medicinal plant belonging to the family of Cephalotaxaceae and no RT-qPCR studies have been reported on it.

**Results:**

In this study, 9 candidate reference genes were selected from the transcriptome data of *C. hainanensis*; 3 statistical algorithms (geNorm, NormFinder, BestKeeper) were applied to evaluate their expression stabilities through 180 samples under 6 stimuli treatments in leaves and leaf-derived suspension cultured cells; a comprehensive stabilities ranking was also performed by RefFinder. The results showed that suitable reference genes in *C. hainanensis* should be selected for normalization relative to different experimental sets. *18S* showed a higher stability than other candidate reference genes which ranked at the top two suitable genes under all experimental setups in this study.

**Conclusion:**

This study is the first to evaluate the stability of reference genes in *C. hainanensis* and supply an important foundation to use the RT-qPCR for an accurate and far-reaching gene expression analysis in *C. hainanensis*.

**Electronic supplementary material:**

The online version of this article (10.1186/s13007-019-0415-y) contains supplementary material, which is available to authorized users.

## Background

*Cephalotaxus hainanensis* is a relic plant which belongs to the genus *Cephalotaxus*, family Cephalotaxaceae. *Cephalotaxus* is the only member of this family, and contains merely 7 species which are all endemic to Asia. It is also a precious medicinal plant and has been listed as endangered species in China. *C. hainanensis* has high medicinal value because of its unique secondary metabolites, cephalotaxine and its derivatives [1–3]. Among the derivatives, *Cephalotaxus* ester alkaloids have anticancer activity with a significant effect in therapy of non-lymphoid leukemia such as acute (slow) myelocytic leukemia, monocytic leukemia, promyelocytic leukemia [4–6]. Especially homoharringtonine has been successfully applied in clinical trials ratified by US food and drug administration (FDA) [7]. However, there is a huge shortage in raw materials of Cephalotaxaceae to produce *Cephalotaxus* ester alkaloids, because Cephalotaxaceae has narrow ecological distribution and grows extremely slow [1, 7]. Although *C. hainanensis* has a higher content of *Cephalotaxus* ester alkaloids, Chinese law has forbidden to obtain its raw materials by cutting the plants. In addition, there is still a lack of a commercially available way of chemical synthesis because of their complicated structure. Hence, genetic engineering and cell engineering to produce *Cephalotaxus* ester alkaloids has become the main research direction and studies on functional genes in their biosynthesis pathway has become the principal issue.

Nowadays, substantial researches on medicinal plants aim to increase their active compounds through inducing and regulating the functional genes in their biosynthesis pathways [8]. Many reports indicated that the synthesis and accumulation of plant secondary metabolites have significant correlation with the expression level of functional genes in their biosynthesis pathways [9, 10]. In these reports, analysis of functional genes by reverse transcription quantitative real-time PCR (RT-qPCR) has become a widely used approach because of its high reproducibility, sensitivity, accuracy and rapidness [11–13]. However, the results of RT-qPCR must to be normalized by reference genes and the stability of reference genes are indeed the critical factor for a reliable result [14, 15]. Many reports indicate that ideal stable reference genes do not exist and the stability of reference genes are varied in different plant species, growth environment, growth stage and stress conditions [16, 17]. Therefore, systematic experimental design for the selection of stable reference genes is the first step towards applying RT-qPCR on a new species. Thus far, stable reference genes have been identified and verified in many plant species including rice [18], potato [19], rhododendrons [20], *Taihangia* flower [21], sugarcane [22], grapevine [23], radish [24], lettuce [25], *Euscaphis konishii* Hayata [26], *Baphicacanthus cusia* (Nees) Bremek [27], creeping bentgrass [28], *Setaria viridis* [29], etc. The reference genes used for these species include *ACT, NAC, UBQ, F*-*box, PP2C, TUA, TUB, UBC, 18S,* and so on. However, the suitable reference genes vary among these plants and even among treatments in same species. At present, the selection and evaluation of reference genes in Cephalotaxaceae remains unreported. It is necessary to perform a multifactorial analysis to identify the stability of reference genes which will greatly facilitate mining the functional genes in *Cephalotaxus* ester alkaloids biosynthesis pathway by comparative transcriptomics.

In this study, nine candidate reference genes (*ACT, NAC, UBQ, F*-*box, PP2C, TUA, TUB, UBC, 18S*) of *C. hainanensis* were selected based on the transcriptome sequencing data by the SMRT (Single-Molecule Real-Time) technology on PacBio Sequel (Unpublished). Six treatments (ABA, Ethylene, Mannitol, MeJA, NaCl, SA) were set to identify their expression level by RT-qPCR in leaves and suspension cells of *C. hainanensis*. The cycle threshold (Ct) values which were detected by RT-qPCR, indicated the expression levels of candidate reference gene directly, lower Ct values represented higher expression levels. Three statistical algorithms (geNorm, NormFinder, BestKeeper) were applied to evaluate their expression stability for normalization. Moreover, comprehensive stability ranking was also performed by RefFinder. Norcoclaurine synthase gene (NCS) catalyzes the first step in the biosynthesis of a diverse class of benzylisoquinoline alkaloids (BIAs) and may also be involved in the biosynthesis of *Cephalotaxus* ester alkaloids in *C. hainanensis.* Therefore, *ChNCS* was selected as the target gene, and its expression was used to verify the reliability of the selected reference genes. Different algorithms were used to screen most stable reference genes and a reliable set of reference genes were provided. Moreover, the criteria for a good reference gene was also discussed.

## Methods

### Plant and suspension cells materials and stress treatments

Plants of *C. hainanensis* were introduced from Jianfeng Mountain, Hainan Province, China. Suspension cells of *C. hainanensis* were generated from tender leaves kept on 0.27% gellan gum (Sigma-Aldrich, Shanghai) containing 4.43 g/L Murashige & Skoog (MS) Basal Medium with Vitamins (PhytoTechnology Laboratories™, U.S.A), 30 g/L sucrose (Sangon, Shanghai), 0.67 µM 6-Benzylaminoprine (6-BA) (Sigma, Shanghai, China), and 1.0 µM 1-Naphthaleneacetic acid (NAA) (Sigma, Shanghai), pH 5.85. Calli were sub-cultured every 5 weeks. Five gram four-week-old calli were transformed into liquid medium (same medium for calli but omitting gellan gum) to create suspension cell culture. The suspension cultured cells were sub-cultured every 10 days by inoculating 5 mL of stationary cells into 30 mL of fresh medium in 100 mL Erlenmeyer flasks. The suspension cells were incubated at 25 °C in darkness on an orbital shaker (Kuhner Shaker, ISF4-X, Germany) at 120 rpm.

In this study, compound leaves excised from five-year-old *C. hainanensis* were used for stress treatments. The leaves were immersed into MeJA (100 μM), SA (100 μM), Mannitol (100 μM) NaCl (100 μM), ABA (10 μM) and ethylene (250 μM) respectively for about 1 s and then placed in a moisture chamber. For suspension cultured cells, the corresponding solutions were added into the medium directly to the indicated concentration. After treatment, leaves and suspension cells were harvested at time point 0 h, 2 h, 6 h, 12 h and 24 h and frozen in liquid nitrogen immediately then stored at −80 °C for RNA extraction. Three biological replicates were prepared for each treatment.

### RNA isolation and cDNA synthesis

Total RNA was isolated from all prepared samples using RNAprep Pure Kit (Polysaccharides & Polyphenolics-rich) (Tiangen, Beijing, China) and genomic DNA was removed with RNase-free DNase I according to the manufacturer’s instructions. The RNA concentration and purity were determined using a Nano Photometer P-Class instrument (Implen, Munich, Germany), and the RNA integrity was also checked on 1% agarose gels. Total RNA (1.0 μg) was used for reverse transcription with a FastQuant RT Kit (Tiangen, Beijing, China) in a 20 μL reaction volume according to the manufacturer’s instructions.

### Primer design and RT-qPCR conditions

Sequences of candidate reference genes (Additional file 5) were mined from our full-length transcriptome database obtained by the SMRT (Single-Molecule Real-Time) technology on PacBio Sequel platform (Unpublished, Novogene, Beijing, China). Specific primer pairs were designed using Beacon Designer 8 software according to primer sequences of 18–24 nucleotides, amplicon length of 75–150 bp, melting temperature (Tm) of 55–60 °C and GC content of 40–60%. All primer pairs were synthesized by a commercial supplier (Sangon, Shanghai, China) and tested by regular PCR and the products were analyzed by electrophoresis on 1.0% agarose gels before RT-qPCR. In addition, amplification efficiency (E) and correlation coefficients (R^2^) were calculated by a standard curve with a series of 5 different cDNA dilutions. The primer sequences, amplicon length, Tm, GC content, amplification efficiency and correlation coefficients of nine candidate reference genes are listed in Table 1.

**Table 1:** Candidate reference genes and target genes description and primer sequences

Gene abbreviation	Gene name	Primer sequences (5′-3′) (forward/reverse)	Amplicon product		E (%) Amplification efficiency	R^2^ Correlation coefficients
			Tm (°C)	Length (bp)		
*ACT*	Actin7	CAGATGTGGATTAGCAAG**/**CAAGCCGTAGTAGGTAAT	79.5	99	92.2	0.988
*UBQ*	Ubiquitin 10	CTTGAGGATGGTCGCACCTT**/**GTCGGAGCTTTCCACTTCCA	83.7	144	98.6	0.998
*NAC*	NAC domain-containing protein	AGGCGTGAAGAAAGTCCTGG**/**CGGTACTCGTGCATGATCCA	82.7	81	98.3	0.993
*F*-*box*	F-box family protein	AAGAGAACAGTGGTAGAC**/**TTGGTAGCAGGATTAAGG	81.8	104	101.3	0.992
*PP2C*	Protein phosphatase 2C	CGACTGATAATGGAATGG**/**CGGAAACTGTAGGTACTA	82.0	123	98.8	0.999
*TUA*	Alpha-tubulin	TCAGTGGATTATGGCAAGA**/**TAGAAGGGAATGGGTTGAC	76.6	114	97.5	0.990
*TUB*	Beta-tubulin	CTCACAGCAATACATAGCC**/**TCAGCAGCACACATCATA	78.8	81	91.1	0.998
*UBC*	Ubiquitin-conjugating Enzyme E2	CTCTACCAATCCACCAGTT**/**GCAAGCCACCAGATAATG	77.8	100	100.9	0.998
*18S*	18S ribosomal RNA	CAATGGCAATGACAATGG**/**CAGAGAACAATCCGAACT	81.7	75	95.1	0.999
*ChNCS*	Norcoclaurine synthase	AGAGGTAATGAGCAATGG**/**TGAGGCGTCTACAAGTTC	82.5	143	98.5	0.996

RT-qPCR was carried out in 384-well plates with a QuantStudio 6 Flex real-time PCR system (ThermoFisher, MA, USA) using SYBR Green-based PCR assay. The final reaction volume for each reaction was 10 μL with the following components: 1 μL diluted cDNA template (1 μg), 5 μL SYBR Premix Ex TaqII (TAKATA, Dalian, China), 1.2 μL forward primer (2.5 μM), 1.2 μL reverse primer (2.5 μM), 1.6 μL ddH_2_O. The reaction was conducted under the following conditions: 95 °C for 7 min, followed by 40 cycles of denaturation at 95 °C for 10 s, and annealing/extension at 56 °C for 30 s. The melting curve was obtained by heating the amplicon from 65 °C to 95 °C with increasing 1.0 °C/s. Each RT-qPCR analysis was performed with three technical replicates.

### Data analysis of gene expression stability

Four statistical tools, geNorm, NormFinder, BestKeeper and RefFinder were used to analyze the candidate reference gene’s stability based on their own algorithms. For geNorm, the expression stability value (M) of each reference gene was calculated based on the average pairwise variation (V) between all genes tested [30]. For NormFinder, an ANOVA-based model of each reference gene was used to calculate the expression stability value by determining inter- and intra-group variation, the gene with the lowest value has the most stable expression [31]. For BestKeeper, the standard deviation (SD) and coefficient of variance (CV) were used to calculate the expression stability of candidate reference genes with raw Ct values, the lowest CV representing the highest stability [32]. For RefFinder (http://150.216.56.64/referencegene.php), a comprehensive ranking was generated with the data from GeNorm (M-values), NormFinder (stability values) and BestKeeper (SD and CV).

### Validation of reference gene stability

Norcoclaurine synthase (NCS) is the first committed enzyme and catalyses a central precursor in the biosynthesis pathway of alkaloids derived from tyrosine and phenylalanine [33, 34]. In this study, we used the *NCS* gene in *C. hainanensis* (*ChNCS*) as target gene to confirm the reliability of the potential reference genes in RT-qPCR. The relative expression level of *ChNCS* under ethylene stress treatment was determined and normalized using the most and least stable reference genes according to the statistical software in the same RT-qPCR conditions mentioned above. The relative expression data was calculated by 2^−ΔΔCt^ method and three technical replicates were performed for each sample.

## Results

### Primers verification and expression levels of candidate reference genes

Primer specificity amplification of all candidate reference genes were verified by regular PCR and RT-qPCR. All primers pairs amplified their specific amplicon based on agarose gel electrophoresis and a single peak with the melting curve analysis (Additional file 1). All candidate reference gene names and abbreviation, accession number, primer sequences, amplification length, efficiency (E) and correlation coefficient (R^2^) are listed in Table 1. Their amplification efficiency varied from 91.1% for *TUB* to 101.3% for *F*-*box*, and correlation coefficients ranged from 0.988 for *ACT* to 0.999 for *PP2C* and *18S*.

**Fig. 1:**
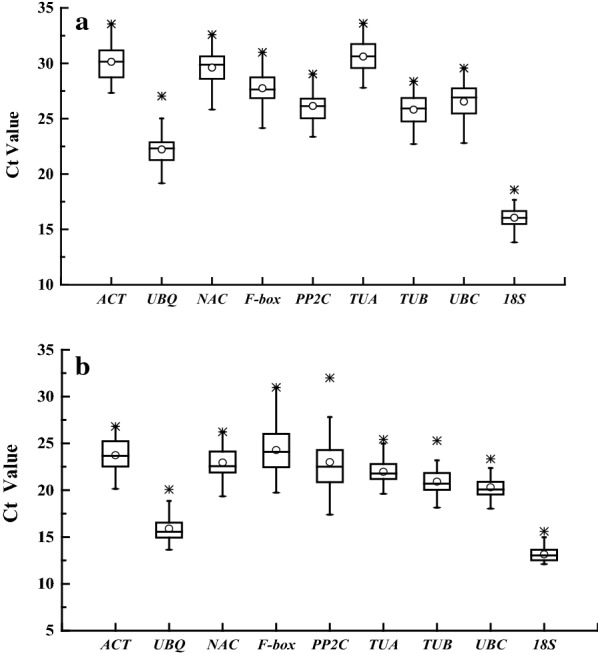
The RT-qPCR Ct values of 9 candidate reference genes across all samples. Expression data displayed as Ct values for each reference gene in *Cephalotaxus hainanensis* leaf (**a**) and cell (**b**) samples. Box graph indicates the 25th and 75th percentiles. The line across the box depicts the median. Lower and upper dashes represent the minimum and maximum values, respectively; middle circle show the mean values. *Represents the extremum value

The expression levels of candidate reference genes were determined by Ct values directly and showed in Fig. 1. The Ct values of all candidate reference genes ranged from 13.83 to 33.61 under different treatments in leaf samples and from 12.11 to 31.99 in suspension cells samples. *18S* had the lowest Ct value both in leaf and suspension cells samples, *TUA* had the highest Ct value in leaf samples and *PP2C* had the highest Ct value in suspension cells samples. In addition, each reference gene had different coefficients of variation (lower values represent less variability) among different conditions. As shown in Fig. 1a, among all leaf samples, the expression of *UBC* (6.00%) varied most and *TUA* (4.44%) least, the others were *18S* (4.63%), *PP2C* (5.19%), *ACT* (5.20%), *NAC* (5.23%), *F*-*box* (5.41%), *TUB* (5.42%), *UBQ* (5.80%). As shown in Fig. 1b, among all suspension cells samples, expression of *PP2C* (12.28%) varied most and *UBC* (5.35%) least, the others were *TUA* (5.45%), *18S* (5.76%), *TUB* (6.07%), *NAC* (6.27%), *ACT* (6.84%), *UBQ* (8.83%), *F*-*box* (11.29%).

## Expression stability of candidate reference genes

### geNorm analysis

**Fig. 2:**
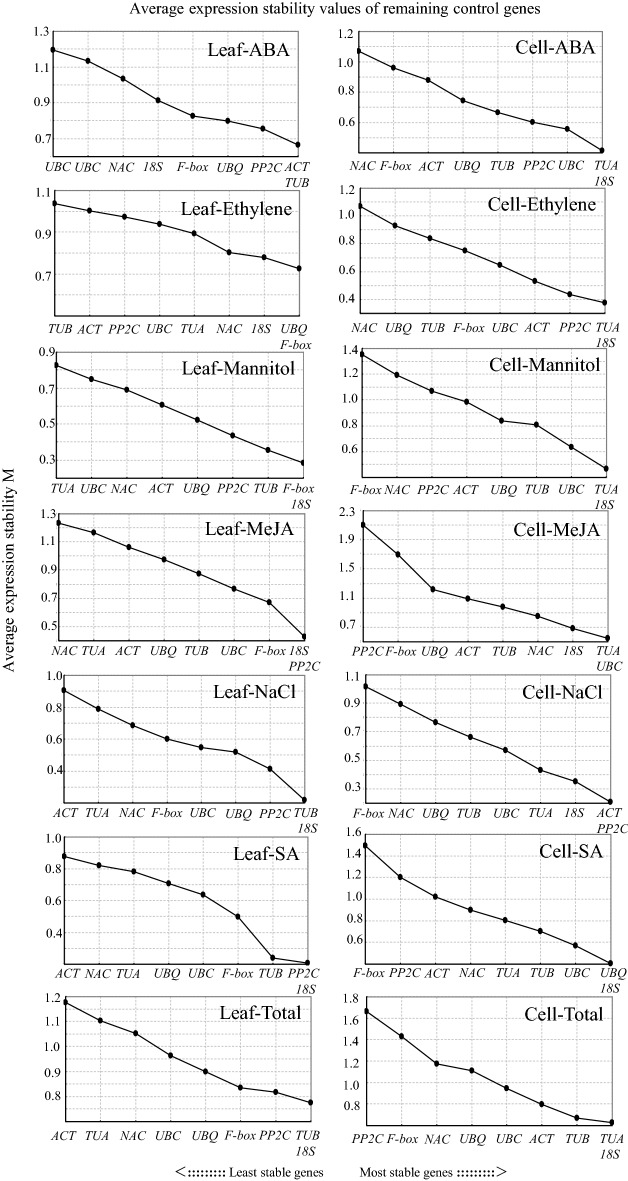
Average expression stability values (*M*) of the 9 candidate reference genes using geNorm software. Expression stability was evaluated in samples from leaf and cell of *Cephalotaxus hainanensis*. submitted to ABA, Ethylene, Mannitol, MeJA, NaCl, SA and Total treatment. The most stable reference genes were measured during stepwise exclusion of the least stable reference genes. The least stable genes are on the left with higher *M*-*value* and the most stable genes on the right with lower *M*-*value*

For geNorm analysis, the stability of all candidate reference genes was evaluated by *M*-*values* below the threshold of 1.5, which were calculated by the mean variation of a gene relative to all others. Lower *M*-*value* represented higher gene expression stability [30]. Based on the geNorm analysis, *M*-*values* were calculated for leaf and suspension cell samples subjected to different treatments respectively. The ranking of the reference genes was found to differ between the different experimental conditions. *18S* and *TUB* had the lowest *M*-*values* and thus were most stable in most leaf treatments; *18S* and *TUA* had the lowest *M*-*values* and thus were most stable in most suspension cells treatments. Contrarily, *ACT* and *NAC* had higher *M*-*values* in most leaf samples and PP2C, F-box and NAC had higher *M*-*values* in most suspension cell samples (Fig. 2). geNorm algorithms also can determine the optimal number of reference genes for normalization calculated by the pairwise variation Vn/n + 1. Ideal pairwise variation (V) score of below 0.15 was recommend. But in this study, most results of pairwise variation calculated by geNorm were more than 0.15 [30] (Additional file 2). Hence, the pairwise variation (V) scores were not suitable. We suggest to use single reference gene to normalize in *C. hainanensis*, because all the *M*-*values* obtained by geNorm were below the ideal threshold 1.5 after all.

### NormFinder analysis

For NormFinder analysis, the stability of all candidate reference genes was evaluated by taking into account intra-group and inter-group variations. The stability values were used to rank candidate reference genes with lower values indicating more stability. As shown in Table 2, the most stable reference genes presented in most leaf samples were *18S* and *UBQ*, the most stable reference genes presented in suspension cells samples were *18S*, *UBC* and *TUA*. Relatively, the least stable reference genes were *TUA*, *NAC* and *ACT* in leaf samples and *NAC*, *F*-*box* and *PP2C* in suspension cells samples.

**Table 2:** Ranking of 9 candidate reference genes according to NormFinder

Rank	Leaf-ABA	Leaf-Ethylene	Leaf-Mannitol	Leaf-MeJA	Leaf-NaCl	Leaf-SA	Leaf-Total
1	*18S*(0.316)	*18S*(0.152)	*PP2C*(0.209)	*UBQ*(0.249)	*TUB*(0.128)	*18S*(0.299)	*18S*(0.267)
2	*PP2C*(0.332)	*F*-*box*(0.262)	*18S*(0.238)	*PP2C*(0.259)	*UBQ*(0.199)	*F*-*box*(0.346)	*UBQ*(0.336)
3	*UBQ*(0.374)	*UBQ*(0.277)	*UBQ*(0.242)	*18S*(0.282)	*UBC*(0.207)	*TUB*(0.421)	*PP2C*(0.356)
4	*TUB*(0.409)	*TUB*(0.287)	*F*-*box*(0.251)	*F*-*box*(0.393)	*18S*(0.252)	*UBC*(0.444)	*F*-*box*(0.363)
5	*ACT*(0.570)	*UBC*(0.298)	*TUB*(0.369)	*UBC*(0.471)	*PP2C*(0.299)	*PP2C*(0.474)	*TUB*(0.392)
6	*NAC*(0.575)	*TUA*(0.354)	*UBC*(0.417)	*TUB*(0.621)	*F*-*box*(0.320)	*UBQ*(0.507)	*UBC*(0.440)
7	*F*-*box*(0.598)	*PP2C*(0.502)	*NAC*(0.455)	*ACT*(0.648)	*TUA*(0.462)	*ACT*(0.570)	*NAC*(0.454)
8	*UBC*(0.613)	*ACT*(0.504)	*ACT*(0.463)	*NAC*(0.687)	*NAC*(0.699)	*TUA*(0.586)	*ACT*(0.489)
9	*TUA*(0.686)	*NAC*(0.611)	*TUA*(0.579)	*TUA*(0.808)	*ACT*(0.927)	*NAC*(0.723)	*TUA*(0.497)

Rank	Cell-ABA	Cell-Ethylene	Cell-Mannitol	Cell-MeJA	Cell-NaCl	Cell-SA	Cell-Total
1	*UBC*(0.230)	*TUA*(0.103)	*18S*(0.277)	*18S*(0.578)	*TUA*(0.214)	*18S*(0.320)	*18S*(0.376)
2	*TUA*(0.285)	*18S*(0.218)	*TUA*(0.300)	*ACT*(0.609)	*18S*(0.246)	*ACT*(0.419)	*UBC*(0.380)
3	*18S*(0.317)	*UBC*(0.254)	*UBC*(0.517)	*UBC*(0.685)	*UBC*(0.333)	*UBC*(0.484)	*TUA*(0.436)
4	*PP2C*(0.344)	*PP2C*(0.392)	*PP2C*(0.539)	*TUB*(0.700)	*PP2C*(0.394)	*UBQ*(0.501)	*ACT*(0.530)
5	*TUB*(0.454)	*TUB*(0.416)	*UBQ*(0.652)	*UBQ*(0.727)	*ACT*(0.403)	*TUB*(0.526)	*UBQ*(0.575)
6	*UBQ*(0.526)	*ACT*(0.483)	*TUB*(0.665)	*NAC*(0.811)	*TUB*(0.502)	*TUA*(0.574)	*TUB*(0.669)
7	*ACT*(0.696)	*UBQ*(0.613)	*ACT*(0.679)	*TUA*(0.825)	*UBQ*(0.595)	*PP2C*(0.789)	*PP2C*(0.673)
8	*NAC*(0.744)	*F*-*box*(0.864)	*NAC*(0.964)	*PP2C*(1.328)	*NAC*(0.708)	*NAC*(0.844)	*NAC*(0.911)
9	*F*-*box*(0.755)	*NAC*(1.103)	*F*-*box*(1.167)	*F*-*box*(1.810)	*F*-*box*(0.861)	*F*-*box*(1.394)	*F*-*box*(0.952)

### BestKeeper analysis

For BestKeeper analysis, the stabilities of candidate reference genes were evaluated using the SD and CV values which were calculated by raw Ct value data directly, lower SD and CV value represented higher gene expression stability, especially when the value SD > 1 indicated the reference gene was unstable and cannot be used for normalization. The results obtained with BestKeeper analysis are shown in Table 3. Among all leaf samples, the most stable reference genes were *18S* for ABA, Ethylene, Mannitol, NaCl and SA treatments, *PP2C* for MeJA treatments and *18S* for total leaf samples; Among all suspension cells samples, the most stable reference genes presented were *18S* for ABA, Mannitol, MeJA and NaCl treatments, *UBC* for Ethylene treatment, *UBQ* for SA treatment and *18S* for total suspension cells samples. Relatively, the least stable reference genes calculated for leaf samples were *F*-*box, ACT, TUB* and *UBC* and for suspension cell samples *F*-*box, ACT* and *PP2C*. Of course, as shown in Table 3, any reference gene with value SD > 1 also presented lower stability and cannot be used for normalization.

**Table 3:** Ranking of 9 candidate reference genes according to BestKeeper

Rank	Leaf-ABA			Leaf-Ethylene			Leaf-Mannitol			Leaf-MeJA			Leaf-NaCl			Leaf-SA			Leaf-Total		
	Gene	SD	CV	Gene	SD	CV	Gene	SD	CV	Gene	SD	CV	Gene	SD	CV	Gene	SD	CV	Gene	SD	CV
1	*18S*	0.37	2.33	*18S*	0.50	3.15	*18S*	0.49	2.97	*PP2C*	0.59	2.38	*18S*	0.40	2.48	*18S*	0.62	3.94	*18S*	0.69	4.30
2	*UBC*	0.75	2.90	*TUA*	0.71	2.29	*NAC*	0.69	2.27	*18S*	0.73	4.53	*TUB*	0.53	2.00	*UBQ*	0.89	4.07	*UBQ*	0.98	4.40
3	*PP2C*	0.96	3.75	*ACT*	0.83	2.74	*UBQ*	0.71	3.22	*UBQ*	0.77	3.68	*UBQ*	0.54	2.37	*NAC*	0.97	3.29	*PP2C*	1.10	4.21
4	*UBQ*	0.96	4.30	*UBQ*	0.92	4.13	*TUA*	0.90	2.87	*ACT*	0.79	1.37	*UBC*	0.55	2.00	*TUA*	0.98	3.25	*TUA*	1.14	3.74
5	*NAC*	1.02	3.48	*UBC*	1.03	3.75	*PP2C*	0.91	3.52	*NAC*	0.97	3.49	*PP2C*	0.64	2.41	*F*-*box*	0.99	3.62	*TUB*	1.15	4.44
6	*TUA*	1.09	3.54	*TUB*	1.06	4.01	*F*-*box*	1.07	3.83	*UBC*	1.05	4.21	*NAC*	0.69	2.23	*TUB*	1.09	4.35	*F*-*box*	1.19	4.30
7	*ACT*	1.18	4.03	*NAC*	1.06	3.49	*UBC*	1.07	3.90	*TUA*	1.05	3.55	*F*-*box*	0.85	2.99	*PP2C*	1.10	4.25	*NAC*	1.25	4.24
8	*TUB*	1.24	4.94	*PP2C*	1.14	4.23	*TUB*	1.12	4.27	*F*-*box*	1.06	4.04	*TUA*	1.05	3.39	*UBC*	1.14	4.35	*ACT*	1.31	4.34
9	*F*-*box*	1.27	4.57	*F*-*box*	1.27	4.52	*ACT*	1.16	3.87	*TUB*	1.11	4.47	*ACT*	1.16	3.74	*ACT*	1.20	4.04	*UBC*	1.32	4.98

Rank	Cell-ABA			Cell-Ethylene			Cell-Mannitol			Cell-MeJA			Cell-NaCl			Cell-SA			Cell-Total		
	Gene	SD	CV	Gene	SD	CV	Gene	SD	CV	Gene	SD	CV	Gene	SD	CV	Gene	SD	CV	Gene	SD	CV
1	*18S*	0.22	1.79	*UBC*	0.46	2.33	*18S*	0.58	4.25	*18S*	0.42	3.28	*18S*	0.24	1.88	*UBQ*	0.27	1.68	*18S*	0.62	4.74
2	*UBC*	0.37	1.87	*18S*	0.46	3.57	*UBQ*	0.66	4.10	*NAC*	0.56	2.29	*UBC*	0.50	2.52	*18S*	0.31	2.23	*UBC*	0.82	4.06
3	*TUA*	0.47	2.18	*UBQ*	0.47	3.04	*TUA*	0.78	3.55	*UBC*	0.63	3.09	*UBQ*	0.61	4.07	*UBC*	0.52	2.48	*TUA*	0.94	4.28
4	*TUB*	0.54	2.67	*TUA*	0.66	3.04	*TUB*	0.97	4.61	*TUA*	1.08	4.94	*TUA*	0.62	2.88	*NAC*	0.59	2.48	*TUB*	1.02	4.87
5	*UBQ*	0.60	3.96	*TUB*	0.84	4.07	*NAC*	1.06	4.45	*UBQ*	1.08	5.85	*TUB*	0.63	3.09	*TUB*	0.78	3.66	*UBQ*	1.07	6.71
6	*PP2C*	0.69	3.41	*NAC*	0.90	3.98	*UBC*	1.17	5.66	*TUB*	1.28	6.01	*ACT*	0.90	3.95	*TUA*	0.95	4.21	*NAC*	1.22	5.31
7	*NAC*	1.04	4.68	*PP2C*	1.06	4.76	*PP2C*	1.27	5.59	*ACT*	1.70	7.02	*PP2C*	0.94	4.32	*ACT*	0.97	4.09	*ACT*	1.38	5.83
8	*F*-*box*	1.04	4.61	*ACT*	1.27	5.43	*ACT*	1.52	6.40	*PP2C*	2.33	8.89	*NAC*	0.95	4.22	*PP2C*	1.56	6.58	*F*-*box*	2.14	8.80
9	*ACT*	1.03	4.44	*F*-*box*	1.40	6.13	*F*-*box*	2.11	9.04	*F*-*box*	3.17	11.86	*F*-*box*	1.42	6.33	*F*-*box*	2.32	9.15	*PP2C*	2.14	9.29

### RefFinder analysis

The RefFinder approach was used to determine the comprehensive rankings of candidate reference genes based on the results of common analysis programs (geNorm, NormFinder, BestKeeper). The comprehensive ranking calculated by RefFinder is shown in Table 4, Additional files 3 and 4. *18S* displayed the highest stability among all leaf and suspension cells treatment samples, its comprehensive ranking was No. 1 in most subsets (Leaf-ABA, Leaf-Ethylene, Leaf-Mannitol, Leaf-SA, Leaf-Total, Cell-ABA, Cell-Mannitol, Cell-MeJA, Cell-NaCl, Cell-Total) and No. 2 in the other subsets (Leaf-MeJA, Leaf-NaCl, Cell-Ethylene, Cell-SA). Relatively, the least stable reference genes were *ACT*, *TUA*, *TUB* and *NAC* for leaf sample subsets and *NAC*, *F*-*box* and *PP2C* for suspension cells sample subsets, respectively.

**Table 4:** Most stable and least stable combination of reference genes based on RefFinder

Experimental treatments													
Leaf-ABA		Leaf-Ethylene		Leaf-Mannitol		Leaf-MeJA		Leaf-NaCl		Leaf-SA		Leaf-Total	
Most	Least	Most	Least	Most	Least	Most	Least	Most	Least	Most	Least	Most	Least
*18S*	*TUA*	*18S*	*TUB*	*18S*	*ACT*	*PP2C*	*NAC*	*TUB*	*ACT*	*18S*	*ACT*	*18S*	*TUA*
*PP2C*		*UBQ*		*PP2C*		*18S*		*18S*		*TUB*		*UBQ*	
												*PP2C*	

Cell-ABA		Cell-Ethylene		Cell-Mannitol		Cell-MeJA		Cell-NaCl		Cell-SA		Cell-Total	
Most	Least	Most	Least	Most	Least	Most	Least	Most	Least	Most	Least	Most	Least
*18S*	*NAC*	*TUA*	*NAC*	*18S*	*F*-*box*	*18S*	*PP2C*	*18S*	*F*-*box*	*UBQ*	*F*-*box*	*18S*	*PP2C*
*TUA*		*18S*		*TUA*		*UBC*		*TUA*		*18S*		*TUA*	
												*UBC*	

### Reference genes validation

**Fig. 3:**
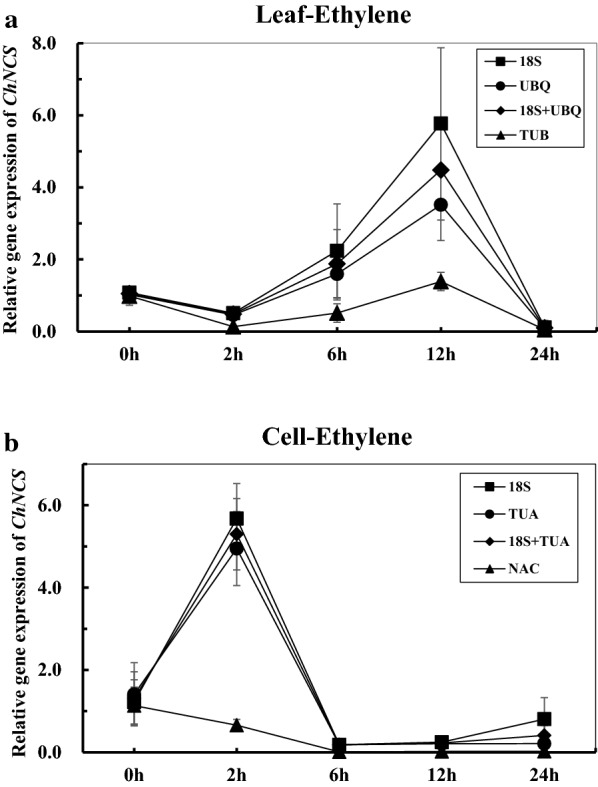
Relative expression of *ChNCS* using selected reference genes including the most or the least stable reference genes for normalization under Leaf-Ethylene and Cell-Ethylene treatment experimental sets. All materials were treated with 250 μM ethylene solution for 0 h, 2 h, 6 h, 12 h and 24 h. The error bars represent standard errors with 3 repeats. **a** Relative expression of *ChNCS* of Leaf-Ethylene treatment; **b** relative expression of *ChNCS* of Cell-Ethylene treatment

The ethylene induced expression level of *ChNCS* in leaf and suspension cell samples was normalized to validate the selected reference genes. in *C. hainanensis*. According to the comprehensive analysis of geNorm, NormFinder, BestKeeper and RefFinder, two sets of reference genes were selected. The most stable reference genes were *18S* and *UBQ* for Leaf-Ethylene samples and *18S* and *TUA* for Cell-Ethylene samples; the least stable genes were *TUB* for Leaf-Ethylene samples and *NAC* for Cell-Ethylene samples. Both in Leaf-Ethylene and Cell-Ethylene samples, the *ChNCS* expression in the 0 h samples were assumed as ‘1’ and we used 2 (^−ΔΔCt^) to calculate its relative expression in samples at other time points. As shown in Fig. 3, results for Leaf-Ethylene samples showed that when the most stable reference genes (*18S* and *UBQ*) were used for normalization, the relative expression of *ChNCS* was 0.51 and 0.46 times higher for 2 h samples; 2.24 and 1.60 times higher for 6 h samples; 5.77 and 3.52 times higher for 12 h samples; and 0.11 and 0.09 times higher for 24 h samples. No large difference was observed in the expression trend of both *18S* and *UBQ* normalization results. However, when the least stable reference genes *TUB* was used for normalization, the relative expression of *ChNCS* showed a different result especially for the 12 h samples, where it is only 1.39 times higher than the 0 h samples. Similar results were observed for the Cell-Ethylene samples, when the most stable reference genes were used for normalization, the relative expression of *ChNCS* was 5.68 and 4.95 times higher for 2 h samples. Conversely, a large difference was evident in the change patterns when the least stable reference gene was used for normalization, the relative expression of *ChNCS* was only 0.66 times higher for 2 h samples.

## Discussion

The endangered *C. hainanensis* harbors unique genes related to medicinally active *Cephalotaxus* ester alkaloids. Quantifying these genes with stable reference genes will greatly facilitate to decipher the biosynthesis pathway. Although leaves have a comparatively lower concentration of interesting metabolites than bark, the fact that it is more renewable and can give a higher biomass makes it a potentially attractive source tissue from this slow growing tree [35]. Moreover, suspension culture cells have shown the potential to produce interesting secondary metabolic compounds by plant cell fermentation techniques [36] and genetic engineering [37]. Therefore, leaves and suspension cultured cells were selected as materials to evaluate the stabilities of reference genes in this study. The stability of reference genes was analyzed comprehensively in *C. hainanensis* under NaCl, mannitol stresses and under challenge of stress-related signaling molecules ABA, ethylene, MeJA and SA. NaCl and mannitol, which have been widely observed to play key roles in the reponse to salt and osmotic stress, can be applied exogenously to mimic such stresses [38].

Previously published studies have pointed out that the stable reference genes varied among species and even changed under different experimental treatments in same species [17]. Since there is still no report on the stability of reference genes in *C. hainanensis*, we selected 9 candidate reference genes for evaluation. By consulting the public studies in other species as well as our transcriptome database, 9 traditional reference genes were extracted as candidates which include protein synthesis (*18S*), cytoskeleton structure (*ACT*, *TUA*, *TUB*), biological metabolic processes (*UBQ*, *UBC*) and novel reference genes of protein phosphatase (*PP2C*), F-box family protein (*F*-*box*), domain-containing protein (*NAC*). All these 9 candidate reference genes had been identified and evaluated in other plant species. In *Baphicacanthus cusia*, *18S* was found to be the most stable gene under ultraviolet irradiation and hormonal stimuli (MeJA and ABA) and *UBC* was the best suitable gene for different plant organs [27]. In *Lycoris aurea*, the comprehensive ranking results presented that *UBC* was the most stable reference gene when challenged by NaCl and cold stress subsets [39]. In *Panax ginseng*, *ACT* was one of top three-ranked genes in seedlings treated with heat [40]. In *Rhododendrons*, *ACT* and *18S* were found to be the top choices for different tissues, whereas *TUB* was not found to favor RT-qPCR normalization in these tissues and *NAC* also was not the suitable reference gene for different tissues [20]. In *Gentiana macrophylla*, *F*-*box, UBQ* and *UBC* were tested as reference genes, but only *UBC* could be considered as reference gene as only the expression of *UBC* was stable enough in multiple tissues and environments [41].

Since the raw Ct values of candidate reference genes are also the direct readout of expression level [16, 28], we then firstly evaluated stability of Ct values in leaf and suspension cell samples. However, the raw Ct values showed large differences under same treatment samples between leaf and suspension cells, which can be seen obviously in Fig. 1, there are approximately 5–7 Ct values in leaf samples more than the same treatment in suspension cell samples. Hence, the leaf and suspension cell samples were separated in the following analysis by geNorm, NormFinder, BestKeeper and RefFinder, which are the most popular statistical algorithms and widely used in recent studies. Numerous reports had confirmed that the ranking results obtained by different statistical algorithms were not completely identical since their different calculation methods [20, 21, 25, 27, 41]. In this study, the similar conclusions were obtained after analysis by statistical software. For example, the top three ranking results under Leaf-Ethylene were *UBQ*, *F*-*box* and *18S* by geNorm algorithms; *18S*, *F*-*box* and *UBQ* by NormFinder algorithms; *18S*, *TUA* and *ACT* by BestKeeper algorithms. Relatively, the three least stable reference genes were also different under Leaf-Ethylene, *PP2C*, *ACT* and *TUB* by geNorm algorithms; *PP2C*, *ACT* and *NAC* by NormFinder algorithms; *NAC*, *PP2C* and *F*-*box* by BestKeeper algorithms. In order to obtain a relatively objective result, the RefFinder software was used for comprehensive ranking and based on this the 18S was identified as the most stable reference gene for *Cephalotaxus hainanensis*. Furthermore, the same analysis results were presented under other leaf and suspension cell treatment sets. In addition, as shown in Table 4, Additional files 3 and 4, the comprehensive ranking demonstrates that *18S* had a higher ranking number and more stable expression level among all the samples.

Many reports indicated that multiple reference genes used for normalization would obtain more accurate results by RT-qPCR and the optimal number could be calculated by geNorm algorithms with ideal pairwise variation (V) score below cut-off value of 0.15 [25, 27]. As shown in Additional file 2, most treatment sets under leaf and suspension cells didn’t result the ideal value (0.15) and most of their V scores were higher than 0.15. However, the *M*-*values*, which can also be calculated by geNorm algorithms to evaluate the stability of single reference gene, were all below the threshold of 1.5. Though the V scores in this study might intuitively present the pairwise variation Vn/Vn + 1 value, the alternative *M*-*values* indicated that the cut-off value 0.15 of V scores must not be considered as the only criterion. With *M*-*values*, it is even possible to obtain accurate results by single reference gene. The golden rules of qRT-PCR have suggested to take at least 4 reference gene to comprise the deviation by single reference gene [15], here, we provide the priority of selection of reference genes, and suggest that even one high ranking reference genes may be a better choice. We validated the selected reference genes with relative expression level of *ChNCS* under ethylene induction. The normalization results in Fig. 3 had shown obviously, the relative expression trend of *ChNCS* had small changes when using the most stable reference genes to normalize both in leaf and suspension cell treatment samples. By contrast, large changes were noted when the least stable reference genes were used for normalization, even a wrong trend were presented in 2 h suspension cell treatment samples. These validation results confirmed the applicability and correctness of the reference genes selected and evaluated in *C. hainanensis*, also indicated stable reference genes selection and evaluation represent a crucial issue for the proper normalization of the RT-qPCR data.

## Conclusion

In conclusion, this study examined the selection and evaluation of 9 candidate reference genes for RT-qPCR normalization under 6 abiotic stresses treatments in *C. hainanensis* leaves and suspension cells. To the best of our knowledge, this work is the first to validate reference genes in *C. hainanensis* for the normalization of the RT-qPCR data. Based on the above results, we recommend 18S as a suitable reference gene for normalizing expression levels in *C. hainanensis*. Other reference genes can be selected as well but which are most suitable differs depending on the tissue and experimental treatment. These selected stable reference genes collectively supply an important foundation to use the RT-qPCR for an accurate and far-reaching gene expression analysis in *C. hainanensis*.

## Additional files

**Additional file 1**. Gene specificity and amplicon size. Melting curves of 9 reference genes showing single peak.

**Additional file 2**. Pairwise variation (V) analysis of the 9 candidate reference genes.

**Additional file 3**. Expression stability ranking of the 9 candidate reference genes in *Cephalotaxus hainanensis* leaf samples.

**Additional file 4**. Expression stability ranking of the 9 candidate reference genes in *Cephalotaxus hainanensis* suspension cells samples.

**Additional file 5**. The sequences of 10 candidate genes in *Cephalotaxus hainanensis*.
