# Supplementary material for: Evaluation of reference genes for normalizing RT-qPCR in leaves and suspension cells of Cephalotaxus hainanensis under various stimuli
Source: Plant Methods. 2019 Mar 26;15:31. doi: 10.1186/s13007-019-0415-y (PMC6434779; doi:10.1186/s13007-019-0415-y)
Supplement: Supplementary file 1 — Additional file 1. Gene specificity and amplicon size. Melting curves of 9 reference genes showing single peak. [file 13007_2019_415_MOESM1_ESM.docx]

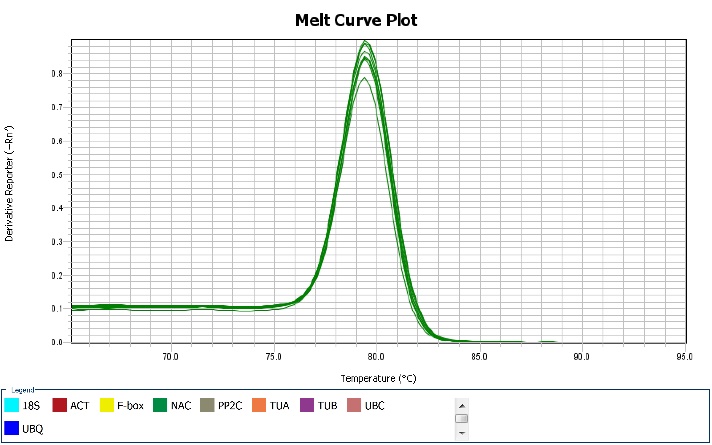


***ACT***


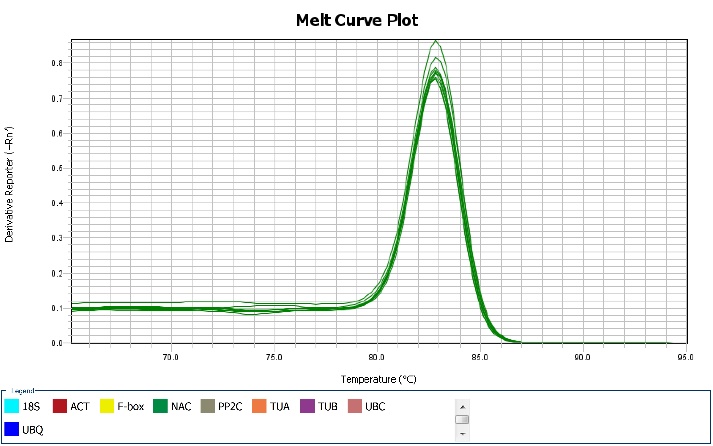


***UBQ***


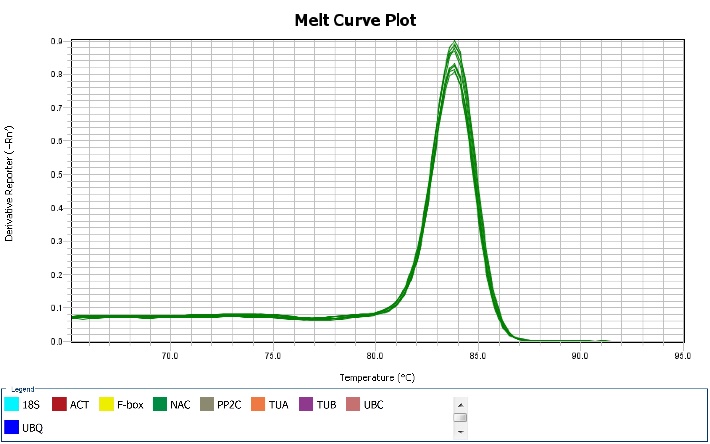


***NAC***


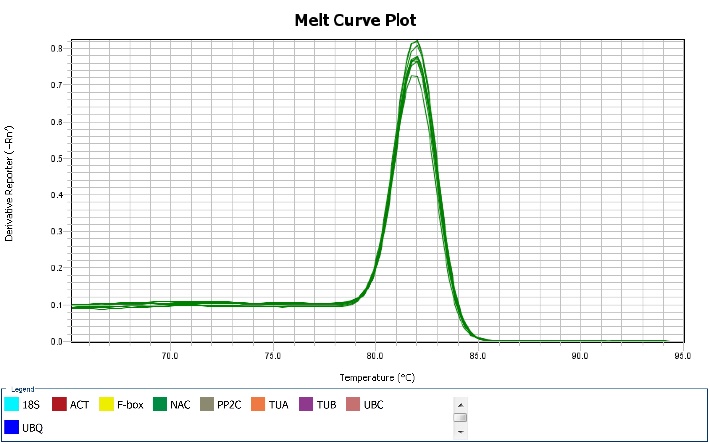


***F-box***


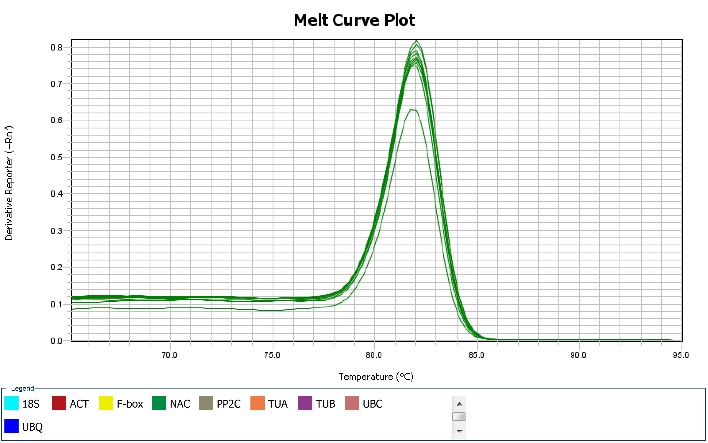


***PP2C***


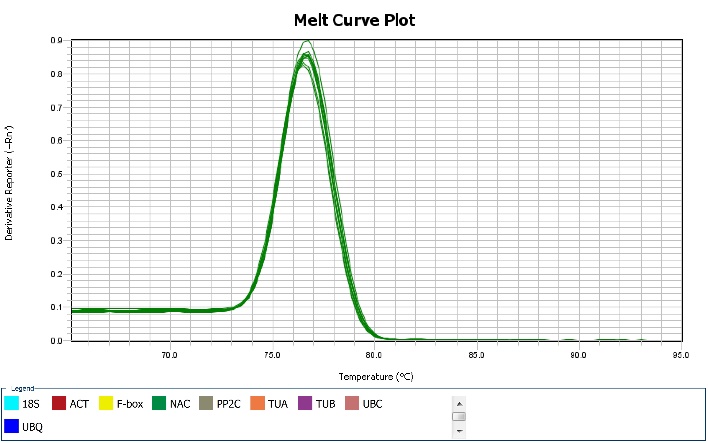


***TUA***


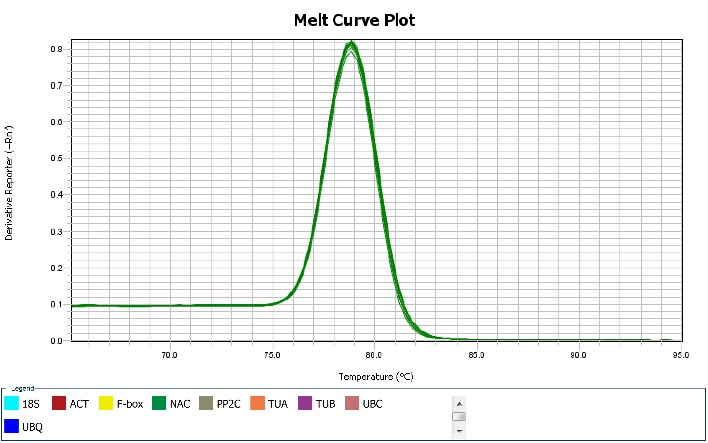


***TUB***


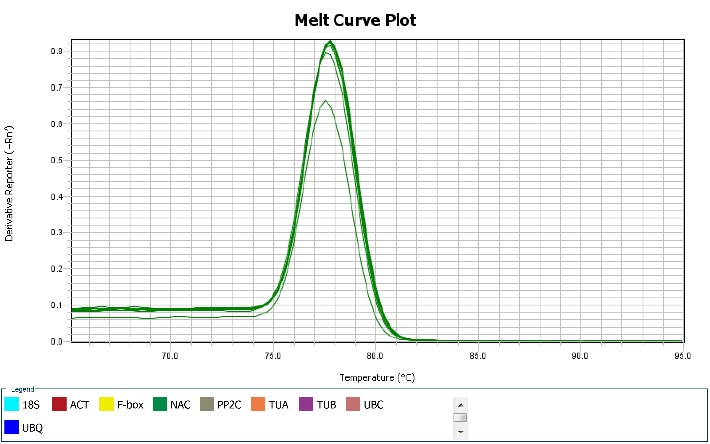


***UBC***


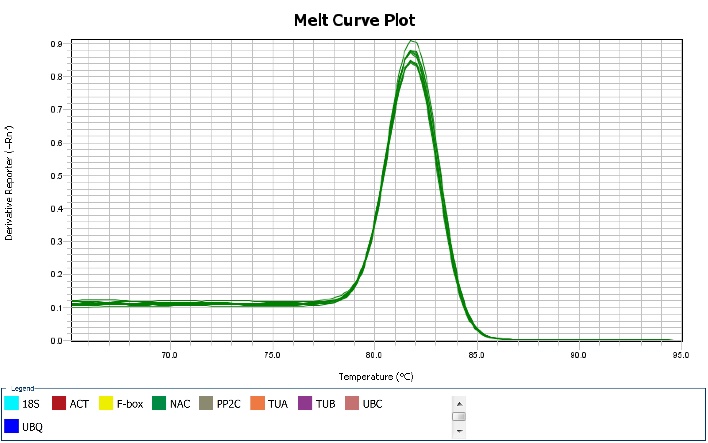


***18S***


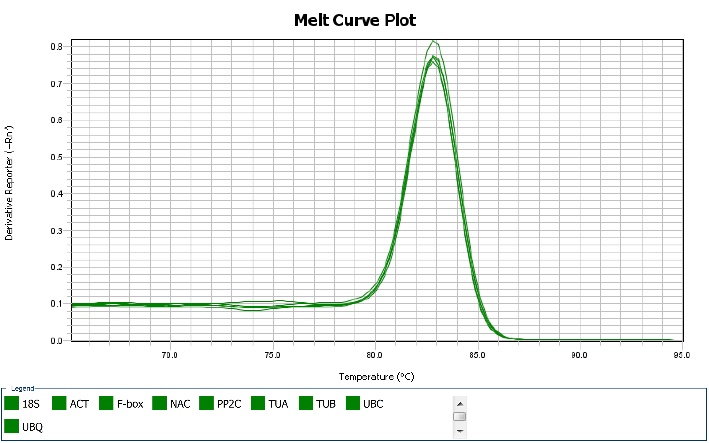


***ChNCS***

**A**


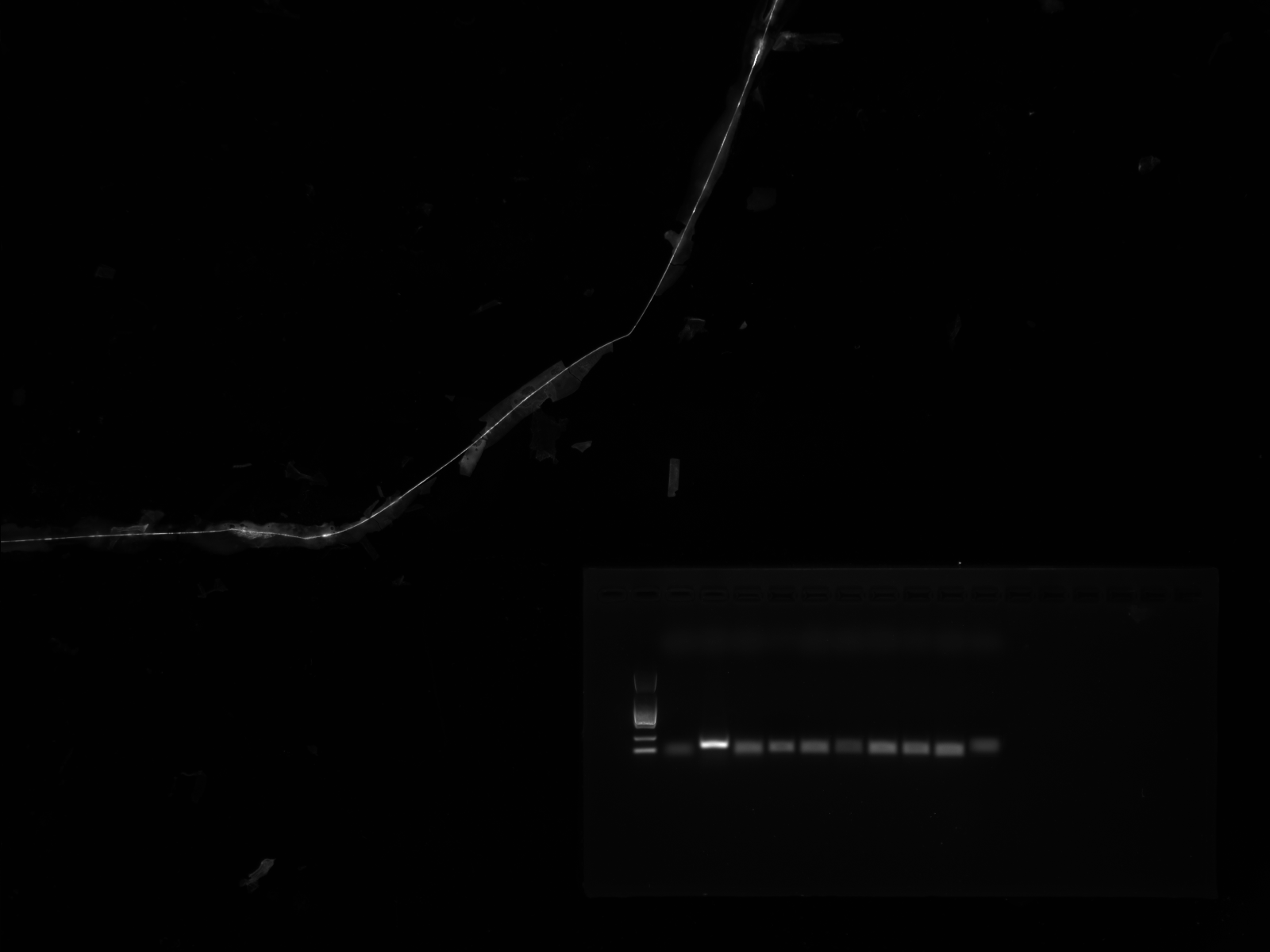


*ACT*

*UBQ*

*NAC*

*F-box*

*PP2C*

*TUA*

*TUB*

*UBC*

*18S*

*ChNCS*

M

100bp

**B**

200bp

Additional file 1 Gene specificity and amplicon size. A: Melting curves of 9 reference genes and *ChNCS* showing single peaks; B: Agarose gel (2.0 %) electrophoresis showing amplification of a specific PCR product of the expected size for each gene.
