# Supplementary material for: Evaluation of reference genes for normalizing RT-qPCR in leaves and suspension cells of Cephalotaxus hainanensis under various stimuli
Source: Plant Methods. 2019 Mar 26;15:31. doi: 10.1186/s13007-019-0415-y (PMC6434779; doi:10.1186/s13007-019-0415-y)
Supplement: Supplementary file 2 — Additional file 2. Pairwise variation (V) analysis of the 9 candidate reference genes. [file 13007_2019_415_MOESM2_ESM.docx]

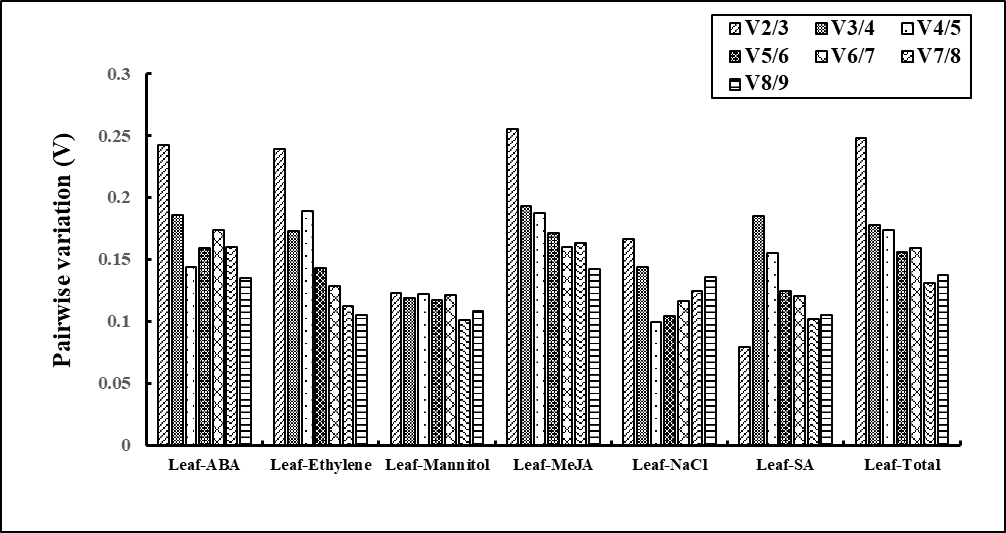


**A**


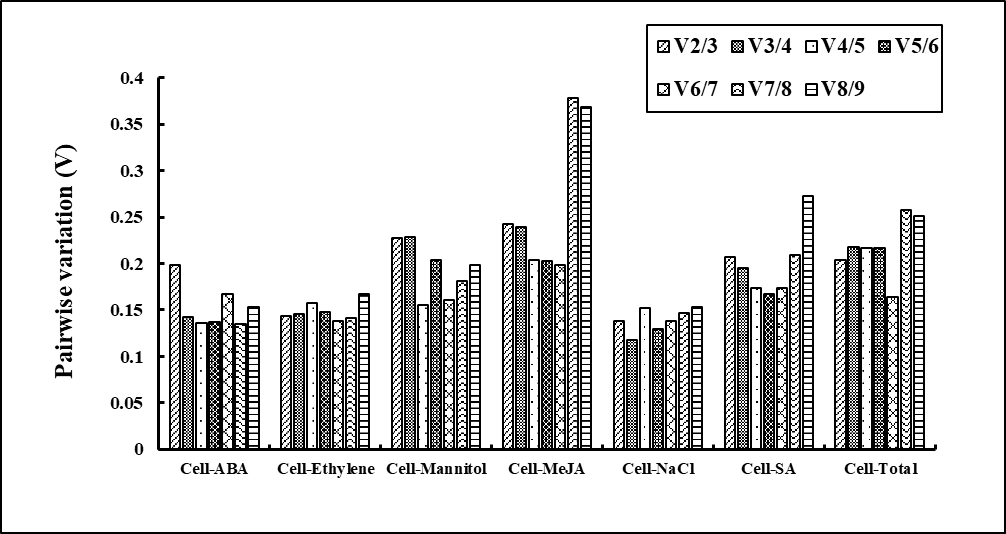


**B**

Additional file 2 Pairwise variation (V) analysis of the 9 candidate reference genes. The pairwise variation (Vn/Vn+1) was analyzed between the normalization factors Vn and Vn+1 by the geNorm software to determine the optimal number of reference genes required for RT-qPCR data normalization.

A: The pairwise variations of leaf were obtained under various stimuli; B: The pairwise variations of leaf were obtained under various stimuli.
