# Supplementary material for: Evaluation of reference genes for normalizing RT-qPCR in leaves and suspension cells of Cephalotaxus hainanensis under various stimuli
Source: Plant Methods. 2019 Mar 26;15:31. doi: 10.1186/s13007-019-0415-y (PMC6434779; doi:10.1186/s13007-019-0415-y)
Supplement: Supplementary file 4 — Additional file 4. Expression stability ranking of the 9 candidate reference genes in Cephalotaxus hainanensis suspension cells samples. [file 13007_2019_415_MOESM4_ESM.docx]

Additional file 4 Expression stability ranking of the 9 candidate reference genes in *Cephalotaxus hainanensis* cell samples

| Method/Rank | 1 | 2 | 3 | 4 | 5 | 6 | 7 | 8 | 9 |
| --- | --- | --- | --- | --- | --- | --- | --- | --- | --- |
| **(A) Ranking Order Under Cell-ABA** | | | | | | | | | |
| BestKeeper | *18S* | *UBC* | *TUA* | *TUB* | *UBQ* | *PP2C* | *NAC* | *F-box* | *ACT* |
| NormFinder | *UBC* | *TUA* | *18S* | *PP2C* | *TUB* | *UBQ* | *ACT* | *NAC* | *F-box* |
| geNorm | *TUA/18S* | | *UBC* | *PP2C* | *TUB* | *UBQ* | *ACT* | *F-box* | *NAC* |
| Comprehensive Ranking | *18S* | *TUA* | *UBC* | *TUB* | *PP2C* | *UBQ* | *ACT* | *F-box* | *NAC* |
| **(B) Ranking Order Under Cell-Ethylene** | | | | | | | | | |
| BestKeeper | *UBC* | *18S* | *UBQ* | *TUA* | *TUB* | *NAC* | *PP2C* | *ACT* | *F-box* |
| NormFinder | *TUA* | *18S* | *UBC* | *PP2C* | *TUB* | *ACT* | *UBQ* | *F-box* | *NAC* |
| geNorm | *TUA/18S* | | *PP2C* | *ACT* | *UBC* | *F-box* | *TUB* | *UBQ* | *NAC* |
| Comprehensive Ranking | *TUA* | *18S* | *UBC* | *PP2C* | *TUB* | *UBQ* | *ACT* | *F-box* | *NAC* |
| **(C) Ranking Order Under Cell-Mannitol** | | | | | | | | | |
| BestKeeper | *18S* | *UBQ* | *TUA* | *TUB* | *NAC* | *UBC* | *PP2C* | *ACT* | *F-box* |
| NormFinder | *18S* | *TUA* | *UBC* | *PP2C* | *UBQ* | *TUB* | *ACT* | *NAC* | *F-box* |
| geNorm | *TUA/18S* | | *UBC* | *TUB* | *UBQ* | *ACT* | *PP2C* | *NAC* | *F-box* |
| Comprehensive Ranking | *18S* | *TUA* | *UBC* | *TUB* | *UBQ* | *ACT* | *PP2C* | *NAC* | *F-box* |
| **(D) Ranking Order Under Cell-MeJA** | | | | | | | | | |
| BestKeeper | *18S* | *NAC* | *UBC* | *TUA* | *UBQ* | *TUB* | *ACT* | *PP2C* | *F-box* |
| NormFinder | *18S* | *ACT* | *UBC* | *TUB* | *UBQ* | *NAC* | *TUA* | *PP2C* | *F-box* |
| geNorm | *TUA/UBC* | | *18S* | *NAC* | *TUB* | *ACT* | *UBQ* | *F-box* | *PP2C* |
| Comprehensive Ranking | *18S* | *UBC* | *TUB* | *TUA* | *NAC* | *UBQ* | *ACT* | *F-box* | *PP2C* |
| **(E) Ranking Order Under Cell-NaCl** | | | | | | | | | |
| BestKeeper | *18S* | *UBC* | *UBQ* | *TUA* | *TUB* | *ACT* | *PP2C* | *NAC* | *F-box* |
| NormFinder | *TUA* | *18S* | *UBC* | *PP2C* | *ACT* | *TUB* | *UBQ* | *NAC* | *F-box* |
| geNorm | *ACT/PP2C* | | *18S* | *TUA* | *UBC* | *TUB* | *UBQ* | *NAC* | *F-box* |
| Comprehensive Ranking | *18S* | *TUA* | *UBC* | *ACT* | *PP2C* | *TUB* | *UBQ* | *NAC* | *F-box* |
| **(F) Ranking Order Under Cell-SA** | | | | | | | | | |
| BestKeeper | *UBQ* | *18S* | *UBC* | *NAC* | *TUB* | *TUA* | *ACT* | *PP2C* | *F-box* |
| NormFinder | *18S* | *ACT* | *UBC* | *UBQ* | *TUB* | *TUA* | *PP2C* | *NAC* | *F-box* |
| geNorm | *UBQ/18S* | | *UBC* | *TUB* | *TUA* | *NAC* | *ACT* | *PP2C* | *F-box* |
| Comprehensive Ranking | *UBQ* | *18S* | *UBC* | *TUB* | *ACT* | *TUA* | *NAC* | *PP2C* | *F-box* |
| **(G) Ranking Order Under Cell-Total** | | | | | | | | | |
| BestKeeper | *18S* | *UBC* | *TUA* | *TUB* | *UBQ* | *NAC* | *ACT* | *F-box* | *PP2C* |
| NormFinder | *18S* | *UBC* | *TUA* | *ACT* | *UBQ* | *TUB* | *PP2C* | *NAC* | *F-box* |
| geNorm | *TUA/18S* | | *TUB* | *ACT* | *UBC* | *UBQ* | *NAC* | *F-box* | *PP2C* |
| Comprehensive Ranking | *18S* | *TUA* | *UBC* | *TUB* | *UBQ* | *ACT* | *NAC* | *F-box* | *PP2C* |
