# Supplementary material for: Evaluation of reference genes for normalizing RT-qPCR in leaves and suspension cells of Cephalotaxus hainanensis under various stimuli
Source: Plant Methods. 2019 Mar 26;15:31. doi: 10.1186/s13007-019-0415-y (PMC6434779; doi:10.1186/s13007-019-0415-y)
Supplement: Supplementary file 5 — Additional file 5. The sequences of 10 candidate genes in Cephalotaxus hainanensis. [file 13007_2019_415_MOESM5_ESM.docx]

Additional file 5: The sequences of 10 candidate genes in *Cephalotaxus hainanensis*.

>Sequence 1 [organism=*Cephalotaxus. Hainanesis*] Actin(ACT) mRNA, complete cds

ATGGAAGACGGCGAGGAAGTGCAAGCTGTTGTGTGTGACAATGGATCTGGCACTGTCAAGGCTGGAATGGCAGGGGACGATGCGCCGAGGGCGGTTTTTCCGAGCATAATCGGACGGCCACGTCACACTGGTGTGATGGTGGGGATGGGGCAGAAGGATGCCTACGTGGGCGACGAGGCGCAGTCGAAGAGGGGTATTCTGACACTCAAATATCCGATCGAGCACGGCGTGGTGTCGAACTGGGATGACATGGAGAAGATCTGGAAGCACACGTTTAGCAACGAGCTGCGCATATCTCCAGAGGAGCACCCCATCCTGCTGACGGAGCCGCCGCTGAATCCAAAGGCCAACAGAGAGAAGATGATCGAGATCATGTTCGACGCATTCAATGTGCCTGCTACCTACATTGCCATCCAGGCAGTGCTCTCCCTCTATGCCAGCGGACGAACAACGGGTATTGTCCTGGATTCAGGAGACGGAGTATCCCACGTGGTGCCCATTTACGAAGGCTACGCTCTGCCGCACGCGATCTGCCGTCTAGATTTAGCCGGCCGTGACTTGACGGCGGCTCTGGCCAAGATCCTAACGGAGCGGGGATATTCTCTAACGACAACCGCGGAACGGGAAATCGTCCGGGACATGAAGGAAAAGCTGGGGTACGTGGCGCTGAATTACGAGCAGGAGCTCCAGATGGCGGCATCGACGCCGTCGCTGGAGAAGAACTACGAATTACCGGACGGGCAGGTGATCACCATCGGGGCGGAGAGGTTCCGGTGCCCGGAGGTGCTGTTTCAGCCGGCACTCATCGGAATGGAAGCTCCGGGGATCCACGAGACGACGTACAACTCCATCATGAAGTGTGACGTGGACATACGTAAAGACCTGTACGGAAACATAGTGATGAGTGGTGGGTCCACCATGTTTCCGGGTATTGCGGACCGCATGCAGAAGGAGGTCTCTGCGTTGGCCCCTCCCTCCATGAAAATAAAGGTGGTGGCTCCTCCCGAAAGAAAGTACAGCGTCTGGATCGGGGGGTCGATCCTCGCTTCTCTCAGCACCTTTCAACAGATGTGGATTAGCAAGGCGGAGTATGATGAATCGGGCTCCTCCATTGTTCATCGGAAGTGTTTTTAG

>Sequence 2 [organism=*Cephalotaxus. Hainanesis*] Ubiquitin 10(UBQ) mRNA, complete cds

ATGCAGATCTTTGTAAAAACCCTTACCGGCAAGACCATTACTCTTGAGGTTGAGAGCTCTGACACCATCGACAATGTCAAAGCCAAGATCCAGGACAAGGAAGGCATTCCCCCAGATCAGCAAAGGCTTATCTTTGCAGGCAAACAGCTCGAAGATGGCCGCACCCTTGCTGACTACAATATCCAGAAGGAGTCCACCCTTCACTTGGTTCTCCGATTGAGGGGAGGGATGCAAATCTTTGTGAAAACGCTCACTGGAAAGACCATCACTCTCGAGGTCGAAAGCTCGGACACCATTGACAATGTCAAGGCCAAGATCCAGGACAAGGAGGGCATTCCCCCAGACCAGCAGAGGCTTATCTTTGCTGGAAAGCAGCTTGAGGACGGTCGCACTTTGGCCGACTACAACATCCAGAAGGAATCGACCCTCCACCTTGTCCTGCGATTGAGAGGAGGCATGCAGATCTTCGTGAAGACTCTCACCGGAAAGACAATTACTTTGGAGGTTGAGAGTTCGGACACTATTGACAATGTCAAGGCCAAGATCCAGGACAAAGAGGGTATTCCCCCAGACCAGCAAAGGCTAATCTTTGCCGGAAAACAACTTGAGGATGGTCGCACCTTGGCGGACTACAACATCCAGAAGGAATCTACCCTCCATCTTGTCCTCCGTTTGAGAGGAGGTATGCAGATCTTTGTCAAGACCCTCACCGGCAAAACCATTACTCTGGAAGTGGAAAGCTCCGACACCATTGATAATGTTAAGGCCAAGATCCAGGACAAGGAAGGTATTCCCCCAGACCAGCAAAGGCTGATCTTTGCTGGAAAGCAGCTTGAGGATGGCCGCACCCTGGCTGATTACAATATCCAGAAGGAGTCTACTCTGCACCTTGTGCTACGTCTCCGTGGTGGTATGCAAATCTTTGTGAAGACCTTGACTGGGAAAACCATTACCCTAGAGGTGGAGAGCTCGGATACCATCGATAACGTGAAGGCAAAGATCCAGGATAAGGAGGGGATTCCACCAGACCAGCAGAGGCTGATCTTTGCTGGTAAGCAGCTTGAGGATGGGCGTACTCTGGCGGATTATAACATCCAAAAGGAGTCGACTCTCCACCTGGTGCTGCGTCTTCGTGGGGGTTTCTAA

>Sequence 3 [organism=*Cephalotaxus. Hainanesis*] NAC domain-containing protein (NAC) mRNA, partial cds

GTTCAGGAAAGGCGAGTTTTGGAGAGAAGGAATGGTATTTCTTCAGCCCCAGAGAGCGCAAGTATCCTAACGGCAACCGCCCCAACAGAGCCGCGGCGTCTGGCTACTGGAAGGCCACCGGCACCGACAAGCCCGTCCTCATATCAGGAACCTCGCGTAGAGCAGGCGTGAAGAAAGTCCTGGTGTTTTACAAAGGCAGACCTCCCAAGGGGGTTAAAACCGACTGGATCATGCACGAGTACCGCCTTACGGACTGTTCCGCCGGGCCGAAACCACCGAGAAAAAAGTGGTCTCTGCGATTGGATGAATGGGTGCTGTGTCGGATTTACCGAAAAGCTGCCCAATTTCCCACCACGGAAAGAAGCCAAGAAGAGGCTTTCATGGAATTGCAGTCGCAGCACAAATTTCAGGTGCCGCCCAAATTTGACTCGTTTTCCGGGGTGGTGCAGAGCTTTCAGAACCCCAACCTCAACTTTATGGGGAGCCTTCTGGATTCGTGGGATGCGAGCGGGGGGGCTGTTGCCGTTCAGGGTCTGCCCAAACTAGACGTCATCTCTACTAAACTGAACTCCTTAAAGAGAAATTCGTCGCTGGTTTTCGATAACGACGATCAAGTCTACTCTCCATCCTTCGATCCCTACAAAGCTGCACTCGCCCATTTACACCAAATTTCTTCGTTGCCCGGCATTGAAAGTGAACACGCCTGGTTATCTTAAACCTGCCTACCTACGATACGATGTTTATCATCAAACTGCCTATTATTTACGACACTACGTTGTTTGCTTAGGTGCAATGATGTAGAGAAG

>Sequence 4 [organism=*Cephalotaxus. Hainanesis*] F-box containing protein(F-box) mRNA, complete cds

ATGCTCAGCCCTGTTCAAATCTGCATGGATTCCTCCGACTGGCCACAGAGCATGATGCAGGAAAGAGGATTTAGGTCTGCTGCTGCTGGTACAGAAGACCTGCTTTCATGTTCACGTCCACTGCTGGAGAGAGGACTGAAACCCCAGCCAGAACAAGCCCTCAAATGCCCAAGGTGCGATTCCCTCAACACCAAGTTTTGCTACTACAACAACTATAGTCTATCTCAGCCTCGCCATTTCTGCAAGACATGTAGAAGGTACTGGACAAAAGGAGGGGCCCTCAGAACTGTCCCTGTTGGTGGGGGTTGCAGGAAGAACAAGAAGGCCAAGAGAACAGTGGTAGACCAGACAGCTTTTTCACAGAATGAGCCATCTACTTCTGGTGCACCACCTTCTGCAAATGCCCTCTCCAACCTTAATCCTGCTACCAATTCAAGCTCCAATATATTTTCTGACATAAATGCTGCTGCTTTTGCCAGAGCCCAACAGCAAGCTGCTGCAAGATATGGAGACCAGTCTGCCTTACCCAACTGCAATAGCTCTGATTTCCTAGGTCTCTCTTGTGGCCCAAGTACTTCCCAACCCTCTTTTGGGCCCCTTACATTAAACCCCATTTCATCACTTACTAATCTGTCAGCACTGAGTACTCTGCAGTCACTGAAATCCAGTTTTCCTGGCCTGACTGATTTCCCTGTGATCAGTAAAAGTACTGATCCGGCATCATCATCATCCCTTGTTCCTCCATCAGATTGGCAACTCCCACCAGAAACCACACTCTTAGACTCCAACATAAACTTCTGGAATGTAGGATCATGGCCTGATCTCCCAACATATGGCTCATCTTCAATGTAG

>Sequence 5 [organism=*Cephalotaxus. Hainanesis*] Phosphatase 2C(PP2C) mRNA, complete cds

ATGGAAGGGCCAGATGTCTGCTGCAGAAGCATAGTGGAACTGGAGAATAAGTTTAGTTCTGCGTGCGAGCGGAAGTCATGTCGAACGGCGAGGCGGAGGCGCATGGAGATCAGAAAGCTTAAGCTCATTGCGACCTCGGGCGTTGTGGAGGCGGCCGCGGCCGCGGCCGAGGCTGCGACAGACGCGGCGGCATCGGTAGGGAGAAAAAGAATCAAGTTATCCAAGGATTCTAGCCACGTTAATGGCAGGGATTGCGTTAACGCCGTCGACAGCTGCACCTTGAAAAACGACATTTCAAAGTCCGGGAATGGAGAAGAAGAGAACGACAGGGTTTCTGCTTCAGCTTCGCCCGTAAAAAATGGCGGAGTGGAGGATAATTTAAGTAACTGTAATGATGATTTCGCGTGCCCGGAGCACGGCATGGTTTCCTTCTGCGGACGGCGTCGGGAAATGGAGGACGCGGTTTCAATTTTACCTGAGTTTAATTGCTCGAAAGATACTCAGCGTAGTTCTGCTTTGCATTTCTTTGGTGTCTATGACGGCCATGGAGGAGCTCAGGCGGCTTTTTTCTGCAAAGATCGGCTCCACGAGGTTCTTGTGGAGGAGCTGAATGCCCGCTGTGATTCACAAAGCGAATTGAAATGGCAGAAGGCGATGGCGAGCTGCTTTAGCAGAGTGGACGCGGAGGTTTTGTCCGGTGGCTTGCGGTGCAAAAATGAATGCAAAGTTAGTGGAATGGGCGAATGCAGGTGCGAGCAGACGATATCGTCGGAAACTGTAGGTACTACCGCAGTCGTTGCTGTTGTTTCCTCACGGCAAATTATCGTCGCTAACTGTGGCGATTCCCGGGCTGTCTTGTCCCGCGGGGGTGTTGCCATTCCATTATCAGTCGATCATAAGCCAGATCGACCAGATGAAATGTCACGTATAGAATCCGCGGGCGGCCGCGTTATCTTTTGGAACGGCCCGCGGGTTTTTGGCGTTCTCGCCATGTCCAGGGCCATAGGCGACAAATACCTGAAGCCTTACGTCATATCGGAGCCGGAGGTGACGATAACGGACCTGACAGACGAAGATGAGTGTCTGATTCTGGCTAGTGACGGACTGTGGGACGTTCTGTCTAATGAAACGGTGTGCGAGGTGGCTCGAAAATGTCTCTCAGGTAGAATACCTGGTGGGGGTTTGAGGCCATTGTTAGATGGCGGAAATGAATCTCCGTCAGCTGCCGCGGCAGCGCTGCTGACGAAATTGGCTCTCGCAAGAGGGAGCGTCGATAATATCAGCGTGGTGGTGGTAGATTTGAGAAGACATGAGAACAGAAGATGA

>Sequence 6 [organism=*Cephalotaxus. Hainanesis*] Alpha-tubulin (TUA) mRNA, complete cds

ATGCCAAGCGACAGAACAGTCGGAGGGGGGGATGATGCGTTCAATACATTCTTCAGCGAGACAGGAGCGGGGAAGCATGTCCCTCGTGCTGTATTCCTTGATTTGGAGCCCACTGTGATTGACGAGGTCAGAACTGGAACCTACAGGCAGCTCTTCCATCCTGAACAGCTCATTAGTGGCAAAGAGGATGCTGCTAACAACTTTGCCCGAGGTCACTATACCATTGGGAAAGAGATTGTTGATCTCTGCTTGGACCGCATCCGAAAGCTGGCTGACAACTGCACTGGGCTGCAAGGTTTCCTTGTATTCAATGCAGTGGGAGGGGGGACTGGCTCAGGCCTGGGATCTCTTCTCCTTGAAAGGCTTTCAGTGGATTATGGCAAGAAATCCAAACTGGGTTTTACTGTGTATCCCTCCCCACAGGTCTCAACCTCTGTGGTTGAGCCTTACAACAGTGTCCTGTCAACCCATTCCCTTCTAGAGCACACTGATGTTGCTATCATGCTTGACAATGAAGCCATTTATGATATCTGCAGGCGCTCCCTTGACATTGAAAGGCCTACTTATACTAATCTCAATAGACTTGTCTCTCAGGTCATCTCCTCCTTGACAGCTTCCCTCCGGTTCGACGGTGCCCTGAATGTGGATATAACAGAGTTCCAGACCAACCTTGTTCCCTACCCCAGGATTCATTTCATGCTTTCCTCCTATGCTCCTGTTATCTCTGCAGAAAAAGCTTACCATGAGCAGCTCTCGGTCTCTGAGATCACAAACAGTGCGTTTGAGCCAGCCTCCATGATGGCCAAATGTGACCCGAGGCATGGCAAATACATGGCTTGCTGTCTCATGTACAGAGGAGATGTTGTGCCCAAGGATGTGAATGCTGCTGTGGGCATTATCAAGACCAAGAGAACCATTCAATTCGTGGACTGGTGTCCCACTGGGTTCAAGTGTGGTATCAACTACCAGCCTCCCACAGTTGTCCCGGGAGGAGACCTTGCCAAGGTACAAAGGGCAGTTTGCATGATTTCAAACAGCACGAGTGTAGCTGAAGTCTTCTCTAGAATTGATCACAAGTTTGATCTCATGTATGCAAAAAGAGCTTTTGTGCATTGGTATGTGGGAGAAGGTATGGAAGAAGGAGAATTCTCTGAAGCCCGGGAGGATCTGGCTGCCCTTGAGAAGGATTATGAAGAGGTTGGAGCTGACTCTACTGAGGGTGAGGGTGAAGATGAAGGCGAGGAATATTGA

>Sequence 7 [organism=*Cephalotaxus. Hainanesis*] Beta-tubulin(TUB) mRNA, complete cds

ATGGATCTGGAGCCAGGGACTATGGATAGTGTTCGGTCGGGGCCGTATGGGCAGATCTTTAGGCCCGATAACTTTGTGTTTGGGCAGTCTGGCGCTGGAAATAATTGGGCGAAGGGGCATTACACCGAGGGCGCTGAGTTGATTGATTCTGTGCTGGATGTTGTCAGAAAGGAAGCGGAGAACTGCGATTGTTTGCAAGGATTTCAAGTGTGCCATTCGCTCGGGGGAGGCACAGGGTCTGGAATGGGTACCCTGTTGATTTCCAAGATCAGGGAAGAATATCCAGATAGGATGATGCTCACATTTTCTGTTTTCCCTTCGCCCAAGGTTTCTGATACAGTTGTGGAGCCCTACAATGCTACGCTGTCGGTTCATCAGCTGGTGGAGAATGCAGATGAATGCATGGTTTTGGACAATGAGGCACTGTATGATATCTGTTTCAGGACTCTCAAGCTAACTACACCCAGCTTTGGTGATTTGAACCACTTGATATCTGCAACCATGAGTGGAGTTACCTGTTGTTTGAGGTTTCCTGGTCAGCTCAACTCTGATCTCCGGAAGCTTGCAGTTAACCTCATCCCCTTTCCGCGTCTTCACTTCTTCATGGTGGGATTTGCACCGCTGACCTCCCGGGGCTCACAGCAATACATAGCCTTGTCAGTTCCAGAGCTCACGCAGCAGATGTGGGATGCCAAAAATATGATGTGTGCTGCTGATCCTAGGCATGGGAGGTACCTCACAGCCTCTGCCATGTTCAGGGGAAAGATGAGCACCAAGGAGGTAGATGAACAGATGATTAATGTGCAGAACAAGAATTCCTCATACTTTGTGGAGTGGATCCCCAACAATGTAAAGTCGAGTGTGTGTGACATCCCTCCTACAGGACTGAAAATGGCTTCCACCTTTGTTGGCAATTCCACTTCCATTCAGGAGATGTTCAGGAGGGTCAGTGAGCAGTTCACTGCAATGTTCAGGAGAAAGGCTTTCTTGCATTGGTACACTGGTGAGGGCATGGATGAGATGGAGTTTACAGAGGCTGAGAGTAACATGAATGATTTGGTTTCTGAGTATCAACAGTATCAAGATGCAACTGCAGATGAGGATGTTTATGAAGAAGAGGAAGAGGAGGGGGCTGCACATGATATGATGTGA

>Sequence 8 [organism=*Cephalotaxus. Hainanesis*] Ubiquitin-conjugating Enzyme E2 (UBC) mRNA, partial cds

AATTGACAAAATCTTTATGTTCCATACCAATACATTGATTTGGTCGTCCCCTAATATAATCAGACATGCTTAAATTAAGACACCAGTAAAACTTGTTTTCTGTTATTAGTCTGTTTCATATATAATGATCTCAAAGTACATATCATTTGCATGTCTTGAAACAATATATACATTTAATGGGAGAAAAAAAGCTTAATATCCAATTTCATTATAAAAAAAACCCTATATAAAGCATATAGACCAAACTTGGGTACTGGAAATGCTGAAATAATATGCATCAATAACCATAGGCAGCATTAACAACAAGTAACAAATACTTTACCACGTGGAAATCATGAGCTATGTGATATCTGCCAAAAATATGTCAGTTGGAATAGCTGAGCTCAATACTTGACAGTAAACTCCTCTACCAATCCACCAGTTTTTGTCGTACCATTCACAAGTAGAATCCCTTATGAAAAGGTTTTTTTGAACTTGTCATCATGGAACCACCATTGAGTATCTTTAGGTGATCTACCATTTCTGCAATTTTTGACATAACGATCATTATCTGGTGGCTTGCTTGGCGGTGGCGCTTGATAACATTGAGAGGATGCTCATGCAGATGGAGCCAACAGTCATGGCTGGTGACCAGGAGTCATAAAGAATATCTAAGCAAATGTGACCGTTGCTGTAAATGTGGGCATGCATAGGAGTGGCTGCAGGAAAACAACCTGTGGGGCTTCCATGGGATAATGCTCGGGAAAATCCACTTGAAGCTGATAGGTTTCATTGTCAAAAAGGGTCCCGGGTGCGCCTGTAACTTCAATGATCCACCTCTGCAAATTGTCAGTTGCTTTGTGCTTGAATCCAGTGGGGGGGTTAGACTGCCATTCAACAAGCTCTTTTGTAGCCTGTTTAAGGCTATCCTGGACAGACCCTTTCTAGGGGTTGAGGTGCTGCTCATTATCGCGATGCAGAAATAATGAGATCGGTCTGTGCTCC

>Sequence 9 [organism=*Cephalotaxus. Hainanesis*] 18S ribosomal RNA (18S) mRNA, partial cds

GATTGATTGATTGATTCTAGTTATATAGAGAGGGGCACCCCTATCCCTCTCACCTCTCCTATAGATTATTCTAGTTATATAGAGAGGGGCACCCCTATCCCCTATCTTGATTGATTGATCGTGGCCAGCCCCATCATCATAGATTGGTGCCGATAGGGGGGGTGTTGAATGTGATGAAAGCTGCTCCACTATATCTATATAGATATAGAGGCGCTGTTCGATGAGCCGCGTAGTATTAGGTAGTTGGTTAGGTAAAGGCTGACCAAGCCAATGATGCTTAGCTGGTCTTTTCGGATGATCAGCCACACTGGGACTGAGACACGGCCCGGACTCCCACGGGGGGCAGCAGTGGGGAATCTTGGACAATGGGCGAAAGCCCGATCCAGCAATATGCGTGGGTGAAGAAGGGCAATTCCGCTTGTAAAGCTCTTTCGTCGAGTGCGCGATCATGACAGGACTCGAGAAGAAGCCCCGGCTAACTCCGTGCCAGCAGCCGCGGTAAGACGGGGGGGGCAAGTGTTATTCGGAATGACTAGGCGTAAAGGGCACGTAGGCGGTGAATCGGGTTGGAAGTGAAAGTCGCCAACAATTGGCGGGTGCTTCGGAACCAATTCACTTGAGTGAGATAGGGGAGAGTGGAATTTCGTGTGTAGGGATCAAATCCGGAGATATACGAAGGAACGCCAAAAGCGAAGGCAGCTCTCTGGGTCTACACTGACGCTGGGGTGCGAAAGCATGGGGAGCAAACAGGATCAGATACCCTGGTAGTCCATGCCGTAAACGATGAGTGTTCGTCCTTGGTCTACCAATCAATATATTGGTCTATACTGGACCGGATCAGGGGCCCAGCTAACGCGTGAAACACTCCGCCTGGGGAGTACGGTCGCAAGACTGAAACTCAAAGGAATTGACGGGGGGCCTGCACAAGCGGTGGAGCATGTGGTTTAATTCGATACTACGCGCAGAACCTTACCAGCCCTTGACATATGAACAAGTGTGCCTGTCCCTAACGGGATGGTGTGAGCGTATGGTTCATACAGGTGCTGCATGGCTGTCGTCAGCTCGTGTCGTGAGATGTTTGGTTAAGTCCTATAACGAGCGAAACCCTCGTCTTGTGTTGCTGGTCAGACACCTTAATATAGAGGGGGGGGTCCACAGTTCTATATAGAGGGAAGCCTTCTATATAGAGGGGGCCCTATATCTATAACTGGGGGCCCATCTTCTCTATGCGGGGAGAGCCCCCTCTCTCTATAAACAGCCCTCTAGAAAGATGGATGGATATAAGGCGAACCACTCGATCTGAGTGAAGAGAATGTCACCAGCAGCTCTGAGTTATATAAATAGGGCGGCCCCCTCTATATATAACAAGGTATAGCCGAGGGCCCATATCTATATATAGGGCTTTGCCATATAGAAGAAATCAATCTTATATAGGTAGGGGAAAGGACTATATATAGATATGGGCCCCTATCCCTATATATAGTAGGCGTAGGCGGCAGCCCGAGCCATATATAGAATAGAGTAGAGGTGCCTCCTATATATATATAATGGGGCCCCTCTCTCCCCTCTATATATAACTATAACTATAACAGCCCTCTATATAGAACTGTGGACCCCCCCCCCACCCCCATATAGATTATATGAGTATGTAACTGGGCCGACGGCCTACTATACTATATATAAGCTGGGCTGGGGGAGAGGTTCGGTTCGGTTCTATATATAGCTGTCTGTGGCGCGATTGATGACACTCCAACCGTACCACTCAGTGCTGCACTCACAAGAGACTGCAGTGAGATGCTGGAGGAAGGTGGGGATGACGTCAAGTCCGCATGGCCCTTATGGGCTGGGCCACACACGTGCTACAATGGCAATGACAATGGGAAGGCCGTAAGGCGGAGCAAATCCAGAAAGGTTGCCTCAGTTCGGATTGTTCTCTGCAACTCGAGAACATGAAGTTGGAATCGCTAGTAATCGCGGATCAGCATGCCGCGGTGAATATTACCCGGCCCTGTAC

>Sequence 10 [organism=*Cephalotaxus. Hainanesis*] Norcoclaurine synthase(ChNCS) mRNA, complete cds

ATGAAGCTACCGAATTCCAAAACCATTTCACTTCCAGTAGTGCAAGAATTGGCAGCCAAGGATCCTAATGCACTGCCTCAAAGGTACATCAGAGGACAGCAAGAAAGGCCAGCCTCCATAACAACAAACAGCAACCACTCTAGTTCTATCCCCACGATAGACATGGCACTTCTGTCTAAAGACCGTGAGTGCAGGCAAGGAGTGATGGAGAAACTTGCATTTGCTTGCCAAGATTGGGGCTTCTTTCAGGTGATGAATCATGGGATCCGTGTGTCTCTGTTGGAGCAAATGAAAGGAGCCGTGAGGGGTTTCTTCCAACTTCCATTGGAAGAAAAACTCAAGTATGGGATGCAAGAGCGTGAAGGCTATGGCCAGGCCTTTGTAATTTCAGACGAACAAAAACTAGACTGGTCAGACATGTTTTATTTGATAACTCTTCCTGAAGATATTAGAAATATGGATTTCTGGCCAACAAGGCCGGTCGATTTCAGGGGGACTGTGAACGAGTATGGCATGGAAACTCAAAAGCTCTCACATGAGCTTTTGAGTCTAATAGCAGAGACCCTGGGTCTCAAAGCTGACAGTTTTATTAATCCAGGAGGAAAGTGGATGCAGGGGACACGAATGAACTACTATCCAAGGTGCTCAAGGCCAGATCTTGTGTTAGGAATAGGCCCTCATTCAGATGCAACAGACATCACAATATTGCTGCAGGATGATGATGAAGTAGGATTACACATACGCAAGGATGAAGAATGGGTTCCTGTTCAACCCATCCCTGGTGCTCTTGTTATCAACATTGGAGATATGGTAGAGGTAATGAGCAATGGAAAATACAAGAGCATTGAACATCTTGCCCTGCCAAACAGTAAGCGGGAACGAATATCAATTGCTACACTTGGCCTTCCCACCAAAGAAGTACAAGTAGGCCCTCACCCTGAACTTGTAGACGCCTCACATCCTCCCTTATACACCACATTTAAACGTGGCGATTTTGACAAGAATTTCTTCCAAAACAAACTAGAACGCAAGTCAGCTCTACAATTTTGCAAAATCCAATCCTTGGCATGA
